# Identification of atopic dermatitis subgroups in children from 2 longitudinal birth cohorts

**DOI:** 10.1016/j.jaci.2017.09.044

**Published:** 2018-03

**Authors:** Lavinia Paternoster, Olga E.M. Savenije, Jon Heron, David M. Evans, Judith M. Vonk, Bert Brunekreef, Alet H. Wijga, A. John Henderson, Gerard H. Koppelman, Sara J. Brown

**Affiliations:** a MRC Integrative Epidemiology Unit, School of Social & Community Medicine, University of Bristol, Bristol, United Kingdom; b Department of Paediatric Pulmonology and Paediatric Allergology, University of Groningen, University Medical Center Groningen, Beatrix Children's Hospital, Groningen, The Netherlands; c University of Groningen, University Medical Center Groningen, Groningen Research Institute for Asthma and COPD, Groningen, The Netherlands; d School of Social & Community Medicine, University of Bristol, Bristol, United Kingdom; e University of Queensland Diamantina Institute, Translational Research Institute, Princess Alexandra Hospital, Brisbane, Australia; f Department of Epidemiology, University of Groningen, University Medical Center Groningen, Groningen, The Netherlands; g Institute for Risk Assessment Sciences, Utrecht University, Utrecht, The Netherlands; h Julius Center for Health Sciences and Primary Care, University Medical Center Utrecht, Utrecht, The Netherlands; i National Institute for Public Health and the Environment, Center for Nutrition, Prevention and Health Services, Bilthoven, The Netherlands; j Skin Research Group, School of Medicine, University of Dundee, Dundee, United Kingdom; k Department of Dermatology, Ninewells Hospital, Dundee, United Kingdom

**Keywords:** Atopic dermatitis, eczema, environmental, genetic, latent class analysis, PIAMA, ALSPAC, AD, Atopic dermatitis, ALPSAC, Avon Longitudinal Study of Parents and Children, *FLG*, Gene encoding filaggrin, LLCA, Longitudinal latent class analysis, OR, Odds ratio, PIAMA, Prevention and Incidence of Asthma and Mite Allergy

## Abstract

**Background:**

Atopic dermatitis (AD) is a prevalent disease with variable natural history. Longitudinal birth cohort studies provide an opportunity to define subgroups on the basis of disease trajectories, which may represent different genetic and environmental pathomechanisms.

**Objectives:**

We sought to investigate the existence of distinct longitudinal phenotypes of AD and test whether these findings are reproducible in 2 independent cohorts.

**Methods:**

The presence of AD was examined in 2 birth cohort studies including 9894 children from the United Kingdom (ALSPAC) and 3652 from the Netherlands (PIAMA). AD was defined by parental report of a typical itchy and/or flexural rash. Longitudinal latent class analysis was used to investigate patterns of AD from birth to the age of 11 to 16 years. We investigated associations with known AD risk factors, including *FLG* null mutations, 23 other established AD-genetic risk variants, and atopic comorbidity.

**Results:**

Six latent classes were identified, representing subphenotypes of AD, with remarkable consistency between the 2 cohorts. The most prevalent class was early-onset-early-resolving AD, which was associated with male sex. Two classes of persistent disease were identified (early-onset-persistent and early-onset-late-resolving); these were most strongly associated with the AD-genetic risk score as well as personal and parental history of atopic disease. A yet unrecognized class of mid-onset-resolving AD, not associated with *FLG* mutations, but strongly associated with asthma, was identified.

**Conclusions:**

Six classes based on temporal trajectories of rash were consistently identified in 2 population-based cohorts. The differing risk factor profiles and diverse prognoses demonstrate the potential importance of a stratified medicine approach for AD.

In clinical practice, atopic dermatitis (AD; eczema) demonstrates a characteristic itchy erythematous rash^1^ but it has a heterogeneous presentation with variations in timing of onset, persistence,^2^, ^3^ distribution, severity, association with allergic sensitization,^4^ and comorbidity with other atopic diseases.^5^, ^6^ Although the classification of eczema cases into atopic and nonatopic forms is commonplace (in part because the underlying etiology of these may be different^4^), the heterogeneity of longitudinal disease course in AD is less well studied. Most AD cases are diagnosed in early childhood and although most resolve during childhood, some persist into adulthood. We hypothesized that divergent temporal disease patterns may be caused by different genetic and environmental etiological mechanisms. Understanding these differences could influence how AD is defined and treated, paving the way for a phenotype-driven, more personalized approach to the management of childhood AD.

AD is a strongly heritable condition. A total of 31 risk loci have been identified in genetic association studies, including 24 loci that were discovered in white European populations.^7^, ^8^, ^9^, ^10^, ^11^, ^12^, ^13^ The cardinal feature of an itchy erythematous rash is central to all case definitions for AD, but large genetic studies have used a broad case definition, including self-reported AD over a wide age range of pediatric and adult patients. This broad case definition has been necessary to allow collection of the large number of cases required for genomewide analyses, but it does not allow for detailed substratification of AD and dictates that such studies are powered to detect variants common across subtypes of disease, while potentially missing variants with more specific effects on subtypes of the disease.

The aim of our study was to investigate the existence of longitudinal subphenotypes of AD and to test whether these findings are reproducible in 2 independent birth cohorts. We used longitudinal latent class analysis (LLCA), a statistical technique that can be used to model potential subgroups within a data set, to identify different longitudinal patterns of disease. We applied LLCA to cohorts from the United Kingdom and the Netherlands from birth to 16 years or 11 years, respectively. We tested each latent class for association with known genetic and nongenetic risk factors for AD and atopic comorbidities, to investigate the existence of distinct subgroups of disease having different etiological and prognostic profiles.

## Methods

### Avon Longitudinal Study of Parents and Children

The Avon Longitudinal Study of Parents and Children (ALSPAC) is a longitudinal population-based birth cohort study of 14,701 children from Avon, United Kingdom. The study protocol has been described previously^14^ and further details are in this article's Online Repository at www.jacionline.org.

Information regarding the presence/absence of rash consistent with AD was extracted from questionnaires completed by the mothers when the children were aged between 6 months and 16.5 years (at 6, 18, 30, 42, 57, 69, 81, 103, 128, 140, 166, and 198 months). At each time point AD was defined as a positive response to 1 of the following questions: “Has your child had an itchy, dry, oozing or crusted rash on the face, forearms or shins?” (at age 6 months); “Has your child had a skin rash in the joints and creases of their body (e.g. behind the knees, elbows, under the arms) in the past 6-12 months?” (18-166 months); “Has your child had an itchy rash which was coming and going for at least 6 months in the past 12 months and confined to the creases of the knees/ankles/elbows or wrists?” (at 16 years).

Nongenetic risk factors were selected on the basis of existing evidence for association with AD^15^ and data availability in the 2 cohorts. Parental history of AD and asthma was parent-reported in questionnaires completed by the parents or guardians. Breast-feeding was coded as a binary variable of never versus any breast-feeding as reported by the mother when the child was 15 months old. Cat exposure was coded as a binary variable of 0 or 1+ cats in the home, as reported by the mother at 8 weeks' gestation. Children were classified as asthmatic at 7 and 13 years if a parent answered “yes” to “Has your child had asthma in the past 12 months?” Total IgE level was measured in venous blood at 7 years and total IgE level of more than 75 kU/L was defined as elevated.

DNA was obtained from blood and genotypes were determined according to methods described in this article's Online Repository at www.jacionline.org. Individuals were categorized into 2 groups: those with and those without any of the 4 gene encoding filaggrin (*FLG*) null mutations tested (ie, *FLG*^−/−^ and *FLG*^+/−^ vs *FLG*^+/+^).^16^ Genotypes for the remaining 23 established (and replicated) European AD risk variants^13^ were combined into a score, with the value representing a sum of the risk alleles carried across the 23 variants.

### Prevention and Incidence of Asthma and Mite Allergy

Prevention and Incidence of Asthma and Mite Allergy (PIAMA) is a Dutch multicenter birth cohort of 3963 children from allergic and nonallergic mothers. The study protocol has been described previously^17^ and further details are in this article's Online Repository at www.jacionline.org.

The International Study of Asthma and Allergies in Childhood–based questionnaires were used to report symptoms of AD between age 3 months and 11 years (3, 12, 24, 36, 48, 60, 72, 84, 96, 132 months). At each time point, AD was defined as a positive response to both of the following 2 questions: “Has your child had an itching rash that was variably present in the last 12 months?” (or ever at 3 and 12 months) and “Was this rash present around the eyes/ears, foreside ankles, inner side knees or inner side elbows?” (also neck at 3 and 12 months).

Parental history of asthma was taken from questionnaires that asked “Have you ever had asthma?” Any versus never breast-feeding was assessed by questions on infant feeding in the questionnaires administered at age 3 months and 1 year. Cat exposure was coded as a binary variable of 0 or 1+ cats in the home at 3 months after birth. Asthma at 7 and 11 years was defined as a parental report of a doctor's diagnosis of asthma at any time and a parental report of asthma in the last 12 months at age 7 and 11 years. Total IgE level was measured in venous blood at age 8 years and a level of more than 75 kU/L was defined as elevated.

DNA was obtained from blood and mouth swabs and genotypes were determined according to methods described in this article's Online Repository. *FLG* genotype categorization and the non-*FLG* genetic risk score were constructed, as for ALSPAC.

### Statistical analysis

LLCA was used to investigate heterogeneity in patterns of AD. As the name suggests, this method is applied in longitudinal settings,^18^, ^19^ where the aim is to identify distinct subgroups in longitudinal multivariate categorical data. This is akin to cluster analysis, but is more appropriate for binary data and allows for assignment based on probability, rather than definitive partitioning of individuals into classes. Starting with a single latent class, additional classes are added until measures that estimate model fit are optimized. Several statistical criteria (including low Bayesian information criterion, Vuong-Lo-Mendell-Rubin likelihood ratio test, and entropy) were assessed to determine the optimal number of classes; full details are given in this article's Online Repository at www.jacionline.org. Model fitting was carried out in Mplus version 7.0.^20^

Model fit was primarily assessed using only those individuals for whom there was no missing AD symptom data. However, to optimize the use of available data and maximize the cohort size, results were compared with analyses that included individuals for whom data were available for 50% or more of the time points studied (≥6 of the 12 time points in the ALSPAC cohort and ≥5 of the 10 time points in the PIAMA cohort). Association analyses primarily focused on this larger (although incomplete) data set, but results were compared with models from the smaller but more complete data set.

Associations of risk factors and comorbidities with the latent classes were tested using a manual implementation of the bias-adjusted 3-step analysis.^21^ This method accounts for uncertainty in class assignment (see this article's Online Repository at www.jacionline.org). Associations with established risk factors (sex, family history of atopy, breast-feeding, presence of pet cat in the household, *FLG* loss-of function mutation, genetic risk score of 23 established white European AD variants) were tested using multinomial regression, whereas atopic comorbidities (asthma at ages 7 and 11 or 13 years; elevated IgE level at age 7 or 8 years) were tested using logistic regression.

## Results

### LLCA in the ALSPAC cohort

**Fig 1:**
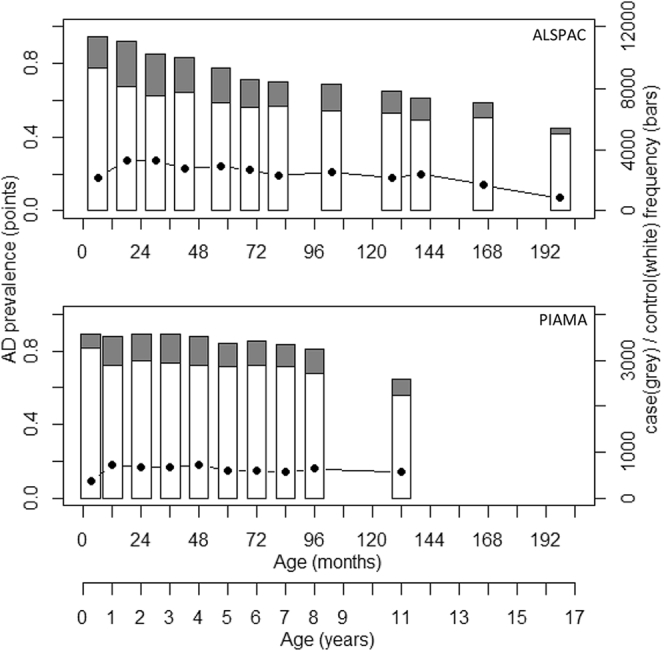
Prevalence and frequency of AD in UK and Dutch longitudinal cohorts. Plot of frequency (right-hand axis) of AD cases (gray bars) and controls (white bars) and AD prevalence (black points and left-hand axis) over 12 time points in ALSPAC and 10 time points in PIAMA. AD is defined by the presence of typical rash.

The prevalence of AD in the ALSPAC cohort declined over time (Fig 1, *A*) from 27% in the first year of life to 7% at age 16.5 years. Data were available from all 12 time points for a total of 3480 individuals. The 6-class model was considered the best fit to the data (as defined by the lowest Bayesian information criterion); however, only small improvements were seen between the 4-class and the 6-class models (see Table E1 and Fig E1 in this article's Online Repository at www.jacionline.org). We present the results of the 6-class model as the primary analysis, but show the results for the simpler 4-class model in this article's Online Repository at www.jacionline.org, which for most analyses produced very similar results.

A total of 9894 individuals had data available from at least 6 of the 12 time points and the model fit parameters were broadly consistent with the smaller but more complete data set (Table E1). Comparison of models from the larger incomplete and smaller but complete data sets showed that the prevalence patterns of AD by class were very similar (see Table E3 in this article's Online Repository at www.jacionline.org) and only 3% of children (116 of 3480) changed best-fit class between the 6-class models in each analysis (see Table E2 in this article's Online Repository at www.jacionline.org).

**Fig 2:**
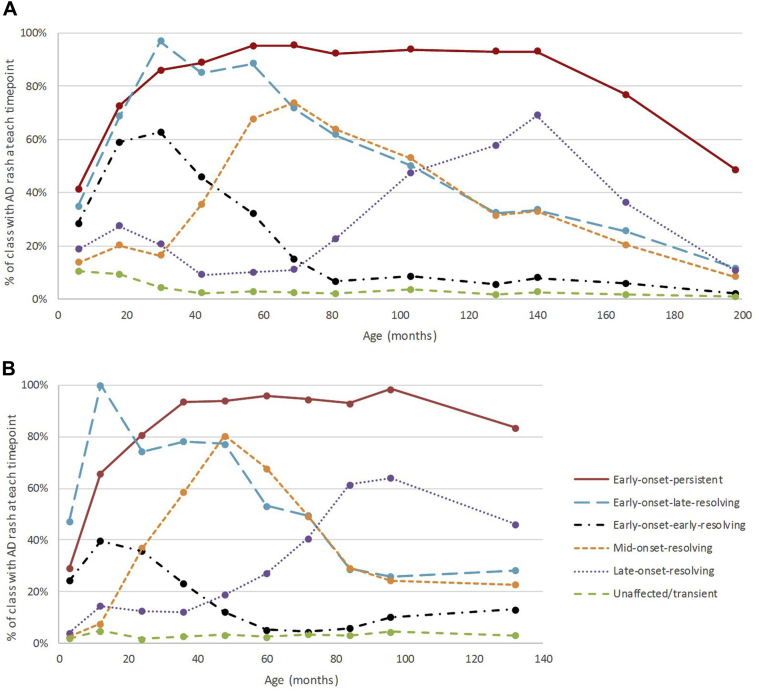
Longitudinal classes identified using LLCA in 2 independent birth cohorts: **A,** ALSPAC (n = 9894) and **B,** PIAMA (n = 3652).

Fig 2, *A*, shows the estimated prevalence of a rash characteristic of AD at each time point across the 6 classes in the analysis of 9894 individuals. Descriptions of the classes alongside the labels we gave each class are given in Table I.

**Table I:** Descriptions and prevalences of the classes in 2 independent cohorts

Class	Description of class in ALSPAC	ALSPAC prevalence	PIAMA prevalence
Unaffected individuals or transient AD	64% of this class never had reported rash, others had 1 or 2 isolated occasions of rash; ∼10% reported rash consistent with AD at 6-18 mo and this declined with age	58.0%	62.9%
Early-onset-persistent AD	At age 30 mo, ∼85% of this class had reported rash, increasing to >90% prevalence until 12 y; it then steadily declined to ∼50% at 16.5 y	7.3%	4.9%
Early-onset-late-resolving AD	In this class the prevalence of rash rose steeply to >95% at 30 mo and then steadily declined to ∼10% by 16.5 y	7.0%	3.8%
Early-onset-early-resolving AD	∼60% of children in this class had reported rash at 18 and 30 mo; this declined to 10% by 6-7 y	12.9%	15.4%
Mid-onset-resolving AD	In this class there was a 10%-20% prevalence of rash until 30 mo, steeply rising to 75% prevalence at 5-6 y, and steadily declining to <10% prevalence by 16.5 y	7.0%	6.5%
Late-onset-resolving AD	In this class, ∼30% reported rash at 18 mo, declining to ∼10% prevalence at 5-6 y, steadily rising to ∼70% prevalence by 12 y and finally declining to 10% by 16.5 y	7.9%	6.5%

**Fig E3:**
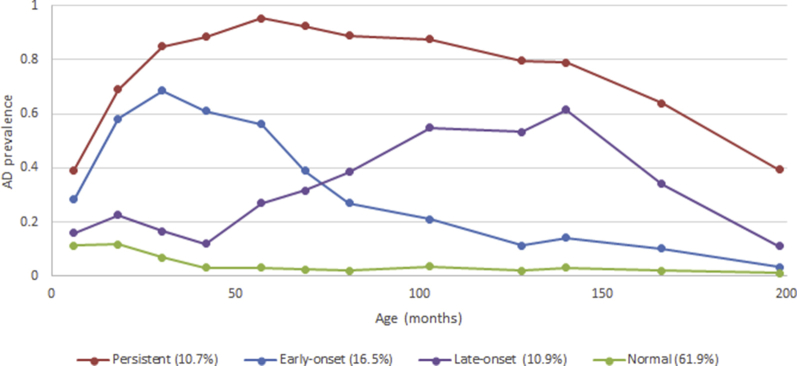


The estimated prevalence of a rash characteristic of AD at each time point for the 4-class model is displayed in Fig E3 in this article's Online Repository at www.jacionline.org. The 4 classes can be described as follows: unaffected individual or transient AD (61.9%); early-onset-persistent AD (10.7%); early-onset AD resolving by age 11 years (16.5%); later-onset AD after age 3.5 years (10.9%). These 4 classes show substantial overlap with the 6-class assignment (see Table E4 in this article's Online Repository at www.jacionline.org).

### LLCA in the PIAMA cohort

**Fig E4:**
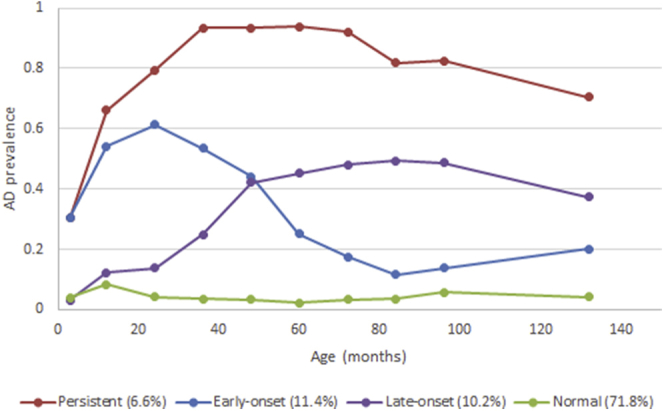


The prevalence of AD in the PIAMA cohort declined only slightly from 18% in the first year of life to 14% by age 11 years (Fig 1, *B*). Data were available from all 10 time points for 2063 individuals. A total of 3652 individuals had data available from at least 5 of these time points, and we present the results from the analysis of this larger incomplete data set. LLCA model fit was similar to ALSPAC, with lowest Bayesian information criteria achieved between the 4-class and 6-class models (see Table E5 and Fig E2 in this article's Online Repository at www.jacionline.org), the resulting class patterns following a remarkably similar pattern to ALSPAC (Fig 2, *B*; see Fig E4 in this article's Online Repository at www.jacionline.org), with comparable class prevalences (Table I and Fig 1, *B*).

As for ALSPAC, we present association results for the 6-class model as the primary analysis because this showed best fit. Comparison of assignment between 4- and 6-class models is shown in Table E7 in this article's Online Repository at www.jacionline.org and association results from the 4-class model are also shown in this article's Online Repository.

### Associations between latent classes with family history and selected environmental risk factors

The associations of 6 classes with potential AD risk factors are summarized in Table II. The results from the smaller but complete data set and the 4-class models are presented in Tables E9 and E10 in this article's Online Repository at www.jacionline.org. Similar conclusions could be drawn from these models, unless otherwise specified.

**Table II:** Association results between risk factors and AD classes identified by LLCA

Trait	Exposed/Total	Wald *P*	Early-onset persistent	Early-onset late-resolving	Early-onset early-resolving	Mid-onset resolving	Late-onset resolving
ALSPAC			7.3%	7.0%	12.9%	7.0%	7.9%
Female	4805/9875	**6 × 10**^**−17**^	**1.56 (1.29-1.89)** ***P* = 6 × 10**^**−6**^	0.97 (0.77-1.22) *P* = .811	**0.75 (0.62-0.91)** ***P* = .004**	**1.79 (1.40-2.29)** ***P* = 4 × 10**^**−6**^	**1.90 (1.48-2.44)** ***P* = 4 × 10**^**−7**^
Maternal eczema	3154/9722	**3 × 10**^**−43**^	**3.16 (2.60-3.83) *P* = 4 × 10**^**−31**^	**1.68 (1.32-2.14)** ***P* = 2 × 10**^**−5**^	**2.00 (1.65-2.44)** ***P* = 4 × 10**^**−12**^	**1.66 (1.29-2.13)** ***P* = 8 × 10**^**−5**^	**1.75 (1.36-2.25)** ***P* = 1 × 10**^**−5**^
Maternal asthma	1554/9721	**2 × 10**^**−4**^	**1.54 (1.22-1.95) *P* = 3 × 10**^**−4**^	**1.43 (1.08-1.91)** ***P* = .014**	1.23 (0.95-1.58) *P* = .112	1.10 (0.79-1.53) *P* = .566	1.02 (0.73-1.44) *P* = .891
Paternal asthma	245/1568	**.030**	1.59 (0.86-2.93) *P* = .139	**2.53 (1.30-4.91)** ***P* = .006**	1.58 (0.86-2.89) *P* = .139	0.94 (0.38-2.33) *P* = .893	1.72 (0.83-3.57) *P* = .146
Breast-feeding	7019/9198	**9 × 10**^**−4**^	**1.42 (1.11-1.81) *P* = .006**	**1.53 (1.12-2.08)** ***P* = .008**	1.22 (0.97-1.54) *P* = .093	1.04 (0.78-1.37) *P* = .803	1.04 (0.78-1.38) *P* = .800
Pet cat	2963/9511	.179	0.88 (0.71-1.09) *P* = .226	1.14 (0.89-1.45) *P* = .291	0.92 (0.74-1.13) *P* = .427	0.81 (0.61-1.06) *P* = .125	1.26 (0.98-1.62) *P* = .073
PIAMA			4.9%	3.8%	15.4%	6.5%	6.5%
Female	1759/3652	**.025**	1.06 (0.75-1.49) *P* = .743	0.63 (0.40-1.00) *P* = .051	0.89 (0.64-1.24) *P* = .494	0.94 (0.65-1.37) *P* = .753	**1.87 (1.21-2.90)** ***P* = .005**
Maternal asthma	259/3645	**.001**	**1.94 (1.11-3.40)** ***P* = .021**	**3.14 (1.76-5.61)** ***P* = 1 × 10**^**−4**^	1.33 (0.70-2.51) *P* = .385	0.96 (0.41-2.24) *P* = .932	1.38 (0.65-2.93) *P* = .406
Paternal asthma	272/3633	**.002**	**2.69 (1.66-4.36)** ***P* = 6 × 10**^**−5**^	0.91 (0.34-2.46) *P* = .854	1.19 (0.63-2.25) *P* = .585	1.72 (0.94-3.14) *P* = .076	1.17 (0.53-2.61) *P* = .697
Breast-feeding	2984/3614	.888	1.00 (0.63-1.56) *P* = .983	1.34 (0.70-2.57) *P* = .377	1.19 (0.75-1.89) *P* = .461	0.97 (0.60-1.56) *P* = .886	0.88 (0.52-1.48) *P* = .634
Pet cat	1213/3651	.151	0.73 (0.50-1.06) *P* = .098	0.80 (0.50-1.29) *P* = .367	0.72 (0.50-1.04) *P* = .081	1.0 (0.68-1.47) *P* = .996	0.67 (0.42-1.07) *P* = .094

“Wald *P*” is for the overall omnibus test. Individual *P* values and effect sizes (OR and 95% CI) comparing each class with the “unaffected/transient” class are also shown; results *P* < .05 are shown in boldface.

In ALSPAC, taking the “unaffected or transient AD” class as the baseline category, being female was a risk factor for the early-onset-persistent, mid-onset, and late-onset classes, the strongest association being with the late-onset class (odds ratio [OR], 1.90; 95% CI, 1.48-2.44; *P* = 4 × 10^−7^). However, male sex was a risk factor for the early-onset-early-resolving class (OR, 1.33; 95% CI, 1.10-1.61; *P* = .004). A similar pattern was observed in PIAMA, where the strongest association with female sex was observed with the late-onset group (OR, 1.87; 95% CI, 1.21-2.90; *P* = .005) and there was evidence of an association between male sex and early-onset-early-resolving class.

Maternal history of AD was associated with all classes in ALSPAC, with the strongest association in the persistent class (OR, 3.16; 95% CI, 2.60-3.83; *P* = 4 × 10^−31^). A similar pattern (albeit with weaker evidence for all classes) was observed in ALSPAC for maternal history of asthma, where again the strongest association was with the persistent class (OR, 1.54; 95% CI, 1.22-1.95; *P* = 3 × 10^−4^). Paternal history of asthma showed a similar association with this class (OR, 1.59), but the smaller sample size meant there was less evidence for this result (*P* = .139). Paternal asthma also showed association with the early-onset-late-resolving class (OR, 2.53; 95% CI, 1.30-4.91; *P* = .006). In PIAMA the associations with maternal and paternal history of asthma were similar, with strong associations with the persistent and early-onset-late-resolving groups for maternal history (OR, 1.94, 95% CI, 1.11-3.40, *P* = .021, and OR, 3.14, 95% CI, 1.76-5.61, *P* = 1 × 10^−4^, respectively) and with the persistent group for paternal history (OR, 2.69; 95% CI, 1.66-4.36; *P* = 6 × 10^−5^).

In ALSPAC, breast-feeding was associated with a higher risk of persistent and early-onset-late-resolving AD (OR, 1.42, 95% CI, 1.11-1.81, *P* = .006, and OR, 1.53, 95% CI, 1.12-2.08, *P* = .008, respectively). There was little evidence of association with mid- or late-onset classes. In PIAMA, there was little evidence for breast-feeding being associated with any class.

Early-life exposure to a pet cat was not associated with any of the latent classes in the primary analyses for ALSPAC or PIAMA. However, this was the only risk factor in which a difference was seen in the complete-case results in ALSPAC, where there was some evidence of a protective effect of early-life cat exposure on the early-onset-early-resolving class only (OR, 0.64; 95% CI, 0.46-0.90; *P* = .010; Table E9). The same direction of effect was observed in PIAMA but with a weaker and less precise estimate (OR, 0.72; 95% CI, 0.50-1.04; *P* = .081).

### Associations between latent classes and atopic traits and comorbidities

The associations of AD classes with elevated total IgE and asthma are displayed in Table III. Raised IgE level was associated with the AD classes showing prevalent disease at the time of testing, that is, age 7 to 8 years (the persistent, early-onset-late-resolving, and mid-onset classes in ALSPAC and the persistent class in PIAMA).

**Table III:** Association results between AD classes identified by LLCA and comorbidities

Trait	Cases/Total	Wald *P*	Early-onset persistent	Early-onset late-resolving	Early-onset early-resolving	Mid-onset resolving	Late-onset resolving
ALSPAC			7.3%	7.0%	15.4%	7.0%	7.9%
Asthma at age 7 y	904/7859	**2 × 10**^**−50**^	**5.50 (4.28-7.05)** ***P* = 5 × 10**^**−41**^	**3.08 (2.22-4.27)** ***P* = 2 × 10**^**−11**^	**1.56 (1.09-2.24)** ***P* = .015**	**2.23 (1.53-3.26)** ***P* = 3 × 10**^**−5**^	**1.89 (1.26-2.83)** ***P* = .002**
Asthma at age 13 y	784/6752	**7 × 10**^**−58**^	**7.19 (5.48-9.42)** ***P* = 3 × 10**^**−46**^	**3.59 (2.51-5.12)** ***P* = 2 × 10**^**−12**^	**1.79 (1.20-2.65)** ***P* = .004**	**3.41 (2.35-4.96)** ***P* = 1 × 10**^**−10**^	**2.01 (1.30-3.12)** ***P* = .002**
Elevated IgE level at age 7 y	2057/4790	**8 × 10**^**−16**^	**2.62 (1.98-3.47)** ***P* = 1 × 10**^**−11**^	**1.92 (1.38-2.68)** ***P* = 1 × 10**^**−4**^	1.15 (0.88-1.51) *P* = .310	**1.55 (1.10-2.18)** ***P* = .013**	1.38 (0.99-1.93) *P* = .059
PIAMA			4.9%	3.8%	15.4%	6.5%	6.5%
Asthma at age 7 y	94/3349	**2 × 10**^**−15**^	**14.27 (7.33-27.78)** ***P* = 5 × 10**^**−15**^	**5.92 (2.31-15.16)** ***P* = 2 × 10**^**−4**^	**3.03 (1.08-8.47)** ***P* = .035**	0.60 (0.03-13.62) *P* = .750	1.73 (0.38-7.88) *P* = .478
Asthma at age 11 y	102/2639	**7 × 10**^**−11**^	**15.35 (6.86-34.35)** ***P* = 3 × 10**^**−11**^	**9.12 (3.49-23.82)** ***P* = 6 × 10**^**−6**^	**4.91 (1.66-14.55)** ***P* = .004**	2.10 (0.42-10.53) *P* = .366	**5.70 (1.98-16.43)** ***P* = .001**
Elevated IgE level at age 8 y	723/1707	**1 × 10**^**−4**^	**3.00 (1.85-4.86)** ***P* = 8 × 10**^**−6**^	1.58 (0.86-2.89) *P* = .140	1.57 (0.97-2.56) *P* = .067	1.42 (0.83-2.46) *P* = .203	0.97 (0.53-1.77) *P* = .914

“Wald *P*” is for the overall omnibus test. Individual *P* values and effect sizes (OR and 95% CI) comparing each class with the “unaffected/transient” class are also shown; elevated IgE level is defined as total IgE level of >75 kU/L; results *P* < .05 are shown in boldface.

In ALSPAC, all classes showed association with asthma at age 7 and 13 years. The associations were strongest for the persistent class (7 years: OR, 5.50, *P* = 5 × 10^−41^; 13 years: OR, 7.19; *P* = 3 × 10^−46^) in which 29% reported asthma at age 7 years (compared with 8% of the normal/transiently affected class), increasing to 31% at 13 years (compared with 7% of the normal/transient class). The early-onset-early-resolving class showed the smallest increased risk of asthma at age 7 and 13 years (ORs, 1.56 and 1.79, respectively). In PIAMA, the persistent and early-onset-late-resolving group showed association with asthma at age 7 years (persistent OR, 14.27; *P* = 5 × 10^−15^). At age 11 years, all but the mid-onset-resolving group were associated, again the strongest association being with the persistent group (OR, 15.35; *P* = 3 × 10^−11^).

### Associations between latent classes and genetic risk variants

In ALSPAC, all classes other than the mid-onset class showed association with *FLG* null mutations (Table IV). The strongest association was for the persistent group (OR, 4.31; 95% CI, 3.29-5.63; *P* = 2 × 10^−26^); the other associated classes had ORs of about half this (2.14-2.30). In PIAMA, only the early-onset-late-resolving class was associated with *FLG* null mutations (OR, 5.63; 95% CI, 2.65-11.95; *P* = 7 × 10^−6^); however, the approximate number of *FLG*^−/+^ or *FLG*^*−/−*^ individuals in the PIAMA analyses was very low (between 7 and 14 individuals per class), so power was limited to identify associations.

**Table IV:** Association results between genetic risk factors and AD classes identified by LLCA

Study and genetic risk factor	No. with risk genotype/total	Wald *P*	Early-onset persistent	Early-onset late-resolving	Early-onset early-resolving	Mid-onset resolving	Late-onset resolving
ALSPAC			7.3%	7.0%	12.9%	7.0%	7.9%
FLG null mutation	813 of 7570	**4 × 10**^**−28**^	131 of 570 (23%)∗	78 of 514 (15%)∗	111 of 832 (13%)∗	54 of 467 (12%)∗	70 of 499 (14%)∗
			**4.31 (3.29-5.63)** ***P* = 2 × 10**^**−26**^	**2.23 (1.53-3.26)** ***P* = 3 × 10**^**−5**^	**2.14 (1.54-2.98)** ***P* = 7 × 10**^**−6**^	1.48 (0.92-2.39) *P* = .109	**2.30 (1.57-3.38)** ***P* = 2 × 10**^**−5**^
Genetic risk score (all other variants)	Total N = 6497	**8 × 10**^**−17**^	**1.17 (1.12-1.22)** ***P* = 2 × 10**^**−13**^	**1.08 (1.04-1.13)** ***P* = 4 × 10**^**−4**^	1.02 (0.99-1.06) *P* = .222	**1.06 (1.00-1.12)** ***P* = .042**	1.01 (0.96-1.06) *P* = .758
PIAMA			4.9%	3.8%	15.4%	6.5%	6.5%
FLG null mutation	117 of 1516	**6 × 10**^**−4**^	7 of 74 (10%)∗	14 of 60 (23%)∗	11 of 159 (7%)∗	7 of 96 (7%)∗	10 of 95 (11%)∗
			1.34 (0.49-3.67) *P* = .563	**5.63 (2.65-11.95)** ***P* = 7 × 10**^**−6**^	0.87 (0.25-3.03) *P* = .821	0.95 (0.26-3.45) *P* = .942	1.87 (0.76-4.62) *P* = .174
Genetic risk score (all other variants)	Total N = 1964	**6 × 10**^**−5**^	**1.17 (1.07-1.28)** ***P* = 5 × 10**^**−4**^	1.0 (0.91-1.11) *P* = .968	**1.16 (1.08-1.25)** ***P* = 1 × 10**^**−4**^	**1.11 (1.03-1.20)** ***P* = .004**	1.08 (0.98-1.18) *P* = .111

“Wald *P*” is for the overall omnibus test. Individual *P* values and effect sizes comparing each class with the “unaffected/transient” class are also shown; results *P* < .05 are shown in boldface; genetic risk score is defined by the total number of risk alleles across the 23 AD-associated loci (other than *FLG*) identified by genome-wide association study meta-analysis to date; OR represents the change in odds per risk allele for the genetic risk score or between carriers compared with noncarriers for the *FLG* mutations.

∗ To demonstrate the approximate numbers of individuals with *FLG* null mutations in each class, individuals were assigned to the most likely class. Given that the actual association analysis accounted for uncertainty in assignment of classes, these values are approximations for purposes of highlighting where power might be low. *The approximate number with FLG null mutations/approximate total with FLG genotype data* (%), within each class are shown. The “unaffected/transient” groups had 8% (∼369 of 4712) and 7% (∼68 of 1032) with *FLG* mutations in ALSPAC and PIAMA, respectively.

The combined genetic risk score encompassing all other AD variants was associated with all but the early-onset-early-resolving and the late-onset classes in the 6-class model in ALSPAC. The association was strongest with the persistent class (OR, 1.17; 95% CI, 1.12-1.22, for each additional risk allele; *P* = 2 × 10^−13^). A similar pattern was observed in PIAMA, with the persistent class showing the strongest association and an almost identical effect size to that seen in ALSPAC (OR, 1.17; 95% CI, 1.07-1.28; *P* = 5 × 10^−4^).

The associations for individual AD risk single nucleotide polymorphism are presented in Table E11 in this article's Online Repository at www.jacionline.org. These analyses are not well powered and should be interpreted with caution, but some patterns are noteworthy. Most variants had the strongest effects in the persistent class and 3 variants showed consistent associations in ALSPAC and PIAMA: These were rs17881320 in *STAT3*, rs479844 near *OVOL1*, and rs6010620 in *RTEL1*. One variant (rs1057258) showed evidence in ALSPAC for association in the opposite direction to that reported previously for AD with the late-onset and early-onset-early-resolving classes (OR, 0.73, 95% CI, 0.57-0.93, *P* = .011, and OR, 0.80, 95% CI, 0.65-0.99, *P* = .039, respectively). A consistent direction of effect (though with weak statistical evidence) was observed for the late-onset class and this single nucleotide polymorphism in PIAMA (OR, 0.74; 95% CI, 0.45-1.20; *P* = .218).

## Discussion

Our results provide novel insights into the heterogeneity of AD in childhood. We report 6 latent classes, representing subphenotypes of AD with remarkable consistency between 2 independent cohorts. The most prevalent class was early-onset-early-resolving AD (13%-15%), which was associated with male sex. This class has a favorable prognosis and is only very weakly associated with asthma in later life. Two classes of persistent disease were identified (early-onset-persistent and early-onset-late-resolving); these were most strongly associated with an AD-genetic risk score as well as personal and parental history of atopic disease. Importantly, these classes display strong comorbidity with asthma. A yet unrecognized class of mid-onset-resolving AD, not associated with *FLG* mutations, but strongly associated with asthma, was described. In this class, AD prevalence increases sharply from age 2.5 years peaking at approximately 6 years. The etiological factors in this class remain unclear because the subgroup was not strongly associated with many of the known risk factors, but does show strong association with asthma comorbidity.

The clinical application of this LLCA is based on the clear demonstration of distinct classes of AD phenotype with different disease trajectories. The substantial diversity of disease that is defined as “AD” (or “eczema”) has long been recognized, and clearer subdivisions are an essential prerequisite for the development of stratified medicine approaches that will be needed for the optimal application of novel biological therapies in the more severe subgroups of AD. Therefore, further studies are needed to define the most appropriate combinations of biomarkers and risk factors to detect these subgroups prospectively and at an early age.

There was some evidence of differential strength and presence of associations with risk factors and comorbidities between the classes. The early-onset-persistent class showed the strongest associations (compared with other classes) with most well-established risk factors and markers of severe atopic phenotype, including *FLG* null mutations and a genetic risk score of other AD-associated variants, coexistent asthma, and elevated IgE and parental history of atopic disease. The associations with asthma at ages 7 and 11 and 13 years were strongest with the persistent class, but all AD classes showed evidence of some increased risk of asthma at these ages. Our data did not support the presence of a specific trajectory from AD to asthma (the so-called atopic march), which is in keeping with a previous report from the Manchester Asthma and Allergy Study (MAAS) and earlier analyses in ALSPAC.^6^ The associations observed with elevated total IgE level were most marked during active and persistent disease, in keeping with previous reports.^22^ Within the class of early-onset disease that resolved before the time of IgE measurement, a smaller proportion of individuals had IgE levels above the threshold defined as “elevated,” in comparison with the class of early-onset disease that was still active. Further investigation with earlier IgE measures is required to explore whether such individuals would have had raised IgE level at the time of active disease.

Although being female was more strongly associated with the early-onset persistent and late-onset classes, there was some evidence that being male was differentially associated with early-onset-resolving classes. It is tempting to speculate that the late-onset class might represent AD induced by behavioral changes in the adolescent child (including increased bathing/showering and the use of fragranced products), which might differ between males and females, but this hypothesis remains to be tested. The male preponderance in AD cases ascertained in infancy has previously been reported^23^, ^24^, ^25^, ^26^ but the mechanisms accounting for this sex difference are unknown.

There is conflicting epidemiological evidence indicating that breast-feeding may be either a risk factor or a protective factor in the etiology of AD^27^ and our analyses have not been able to add clarity to this important question. In the ALSPAC cohort, breast-feeding was associated with the 2 classes of most long-lasting disease (early-onset-persistent and early-onset-late-resolving AD) but in the PIAMA cohort there was little evidence of breast-feeding being associated with any of the latent classes. This apparent difference could be explained in several different ways: It may be stochastic (given that all 95% CIs overlap between the 2 cohorts); it may be a result of the substantially higher prevalence of breast-feeding in the Dutch population compared with the UK population; or it may result from reverse causation in the ALSPAC cohort, if, for example, mothers with a strong history of atopic disease are more likely to breast-feed their infants.

We found little evidence for early-life exposure to a pet cat being associated with any of the classes in the main analysis. However, in the complete-case data set in ALSPAC, there was some evidence that cat exposure may have a protective effect on early-onset-early-resolving AD, which is somewhat at odds with previous reports of cat exposure increasing the risk of AD.^28^, ^29^ This may indicate a specific beneficial effect of early cat exposure on this more transient phenotype and warrants further investigation.

There was evidence that *FLG* null mutations were associated with all classes; however, as reported previously,^30^, ^31^ the association was strongest in the group with early-onset-persistent disease. The genetic risk score of the other established AD variants showed a similar pattern, whereby the association was strongest for the early-onset-persistent class, with a striking increase in the burden of risk of approximately 17% per additional risk allele. Of note, 3 variants showed consistent patterns of effects across both cohorts, with stronger associations in the early-onset persistent group, and weaker associations with the other classes. The functional mechanisms of these loci have not been fully defined, but rs17881320 is within *STAT3* (encoding a signal transducer and activator of transcription, an acute-phase response protein); rs479844 is near to *OVOL1* (which encodes zinc-finger containing transcription factor); and rs6010620 is within *RTEL1* (regulator of telomere elongation helicase 1). This heterogeneity of effect of genetic variants on different disease profiles emphasizes the need for patient stratification in future genetic studies. Stratification may be used to increase the power to detect variants associated with specific classes; stratification could also allow the identification of phenotype-specific mechanistic pathways as future therapeutic targets.

The similarity and high frequency of AD symptom ascertainment in ALSPAC and PIAMA are strengths of our study. The phenotype definitions used within the cohorts comprised prospective questions to capture diagnostic features of eczema including the changing distribution of skin involvement from infancy to later childhood. One key difference is that PIAMA had a shorter follow-up (11 years vs 16 years), which could have limited the ability to detect classes with differences at later ages. Despite this, the class patterns are remarkably similar between ALSPAC and PIAMA (Fig 2). However, neither cohort allowed for investigation of variable AD patterns in adulthood, more subtle AD patterns (such as the transient cases that were indistinguishable from the unaffected individuals in our study), nor in people of ancestries other than white European. We also studied only a limited number of environmental factors and so this work could be extended to investigate the association between latent classes and other potential risk factors.

Although individuals are not assigned to classes with complete certainty (see Table E8 in this article's Online Repository at www.jacionline.org), the LLCA 3-step method models this uncertainty and allows for the inclusion of individuals with incomplete data to maximize sample size and minimize any loss to follow-up bias. We note that our analysis does not formally test for causal direction and some risk factors studied do not entirely precede the onset of disease. Therefore, although the observational associations are interesting, further work should be conducted to investigate causality. A further challenge is that by stratifying AD into smaller subphenotypes of disease, we inevitably lower the power of association testing. Few studies have such detailed longitudinal data and so to increase sample sizes in future studies, it will be necessary to extrapolate these data-driven phenotypes into settings where less detailed data are available, such as large data registries.^32^

In conclusion, we have identified longitudinal subgroups of AD that have both shared and distinctly different risk factor profiles. Future studies of the etiology and treatment of this complex trait should take these subgroups of disease into account and, in turn, this may offer valuable stratified medicine approaches to refining prognostic predictions and therapeutic strategies.

**Clinical implications**

AD ranges from a transient condition to lifelong morbidity. This study has identified distinct subphenotypes of AD in children, which could indicate the importance of a stratified approach to the management of this complex disease.

## Figures and Tables

**Fig E1A:**
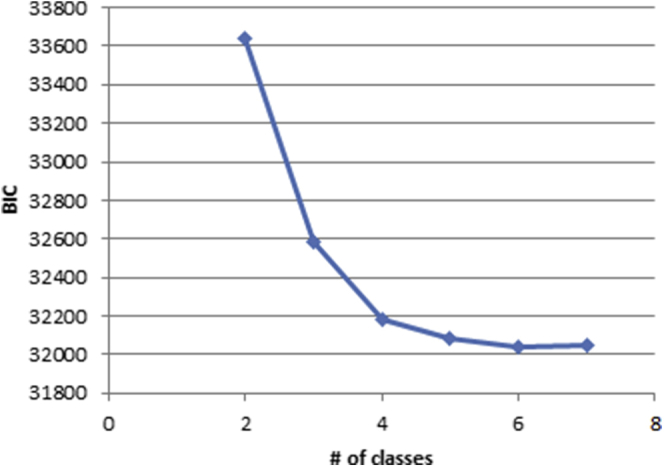


**Fig E1B:**
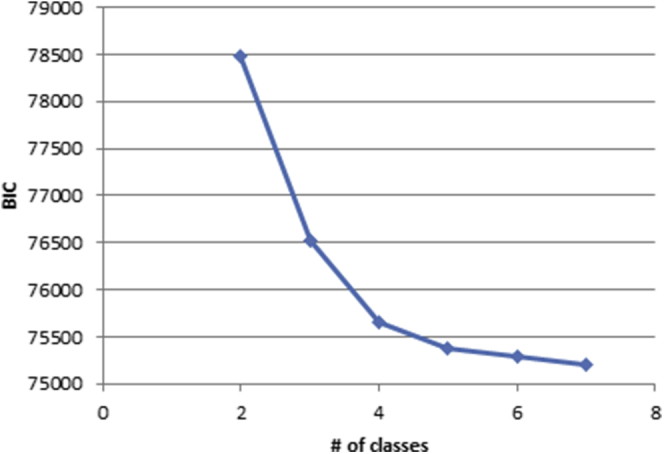


**Fig E2A:**
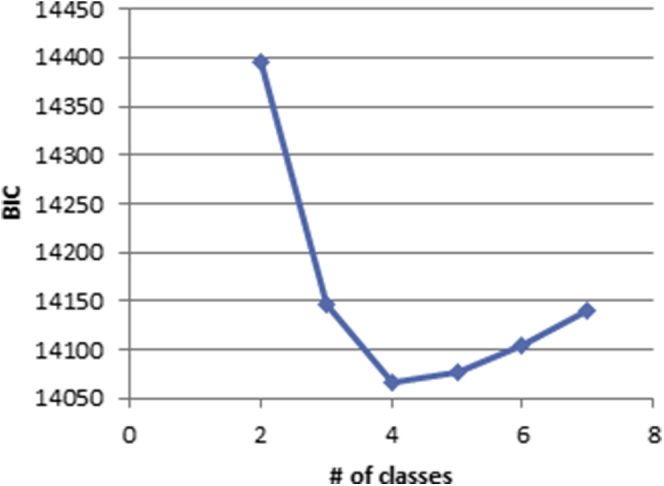


**Fig E2B:**
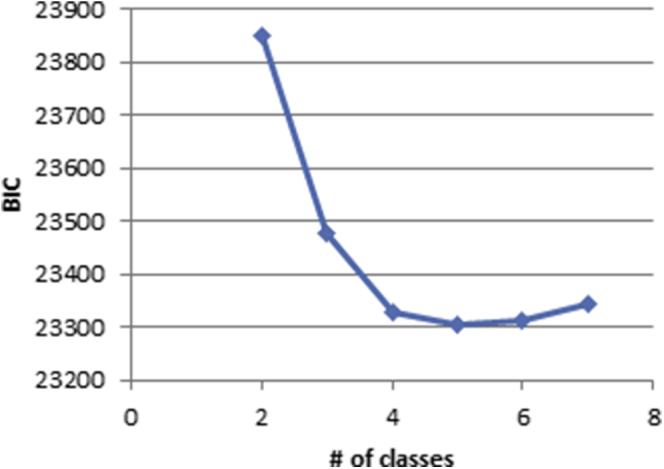


## Supplementary data

Online Repository text and Tables E1-E8

Table E9

Table E10

Table E11
