# Supplementary material for: Identification of atopic dermatitis subgroups in children from 2 longitudinal birth cohorts
Source: J Allergy Clin Immunol. 2018 Mar;141(3):964–71. doi: 10.1016/j.jaci.2017.09.044 (PMC5840507; doi:10.1016/j.jaci.2017.09.044)
Supplement: Online Repository text and Tables E1-E8 [file mmc1.docx]

**Online Repository**

The Avon Longitudinal Study of Parents and Children (ALSPAC)

This population-based birth cohort recruited 15,247 pregnant women resident in Avon, UK with expected dates of delivery from 1st April 1991 to 31st December 1992, resulting in 14,775 live births and 14,701 children who were alive at 1 year of age. Enrolment is described in detail in the cohort profile papers^1,2^ and the study website contains details of all the data that are available through a fully searchable data dictionary^3^. Biological samples including DNA have been collected for 10,121 of the children from this cohort. Ethical approval for this study was obtained from the ALSPAC Ethics and Law Committee and the Local Research Ethics Committees; written informed consent was provided by the parents/guardians.

DNA was obtained from blood samples using standard extraction techniques. The 4 most prevalent loss of function mutations in the gene encoding filaggrin (*FLG*) R501X, 2282del4, R2447X and S3247X were genotyped using KASP^TM^ genotyping technology^4^ (LGC Genomics, Hoddesdon, Hertfordshire, UK). Genome-wide genotyping was undertaken in 9912 children using the Illumina HumanHap550 quad SNP genotyping platform by 23andMe (Mountainview, California, USA), subcontracting to the Wellcome Trust Sanger Institute (Cambridge, UK) and the Laboratory Corporation of America (Burlington, NC, USA). Genotypes were imputed with Impute2 using 1000 Genomes phase 1 version 3 as a reference set as previously described^5^.

Prevention and Incidence of Asthma and Mite Allergy birth cohort study (PIAMA)

The PIAMA study is a Dutch multicenter birth cohort that invited allergic and nonallergic pregnant women to participate; 4146 (53%) agreed and provided written informed consent (1327 allergic and 2819 nonallergic subjects). 183 were lost to follow-up before any data were obtained, so the study started with 3963 new-borns. Parents were sent International Study of Asthma and Allergies in Childhood–based questionnaires about their child’s health, including asthma and eczema symptoms at 3, 12, 24, 36, 48, 60, 72, 84, 96 and 132 months after birth. All high-risk children and a sample of low-risk children were invited for clinical examinations at 4 years of age and at 8 years all children who were still in the study were invited. Full details of the study have been published previously^6^. The study protocol was approved by the medical ethics committees of the participating institutions and informed written parental consent was obtained for each participant.

Blood samples were collected from the children at age 4 and 8 years for DNA extraction. Children who did not participate in a clinical examination were invited to send a buccal swab by mail, and DNA was extracted from buccal swabs as described previously^7^. DNA was obtained from 2,162 children. Genome-wide genotyping was performed in three phases. The first phase was performed within the framework of the GABRIEL Consortium using an Illumina Human 610K quad array^8^. Genotypes were available from 172 children with asthma and from 187 controls after quality control. A second group of 268 children who were more extensively examined during follow up were genotyped with the Illumina HumanOmniExpress array. A final group of 1377 children was genotyped with the Illumina Human Omni Express Exome Array. SNPs were harmonized by base pair position annotated to genome build 37, name and annotation of strand for each platform. Discordant or duplicate SNPs or SNPs that showed large differences in allele frequencies (>15%) were removed. After quality control, a total of 1968 individuals remained and imputation was performed per platform using IMPUTE 2.07 against the reference data set of the CEU panel of the 1000 Genomes project (version March 2012). SNPs of high quality (info-score IMPUTE ≥ 0.7) were merged into one dataset using GTOOL and used for further analysis. Dosages of imputed SNPs were predicted based on the following assumptions; 0 is the first homozygote, 0.5 is a heterozygote and 1 is the other homozygote. The info scores of the imputed SNPs were all ≥0.99 which provided reliable dosages estimates. From this dataset, 22 genotypes were available to calculate genetic risk scores for further analysis. Prevalent *FLG* loss-of-function mutations were genotyped at LGC Genomics as described above for the ALSPAC cohort.

LLCA – selection of best model

Longitudinal latent class analysis was carried out in Mplus. Models with between 2 and 7 classes were tested. Bayesian Information Criterion (BIC), entropy, and p-values from the Vuong-Lo-Mendell-Rubin (VLMR) likelihood ratio tests were compared to ascertain the models with the best fit.

ALSPAC – model fit

In the dataset of complete data (N=3480), the BIC (which is penalized for model complexity) was lowest for a 6 class model (Table E1 and Fig E1a) and the VLMR LR p-value showed evidence of improved fit for the 6-class model compared to a 5-class model (p=0.018). Entropy remained above 0.8 in all models, indicating good class delineation and individual class classifications were all >0.75, with <0.11 for all off-diagonals. The largest decrease in the BIC were seen from 2 to 4 classes (Fig E1a), with only small improvement in model fit between the 4- and 6-class models.

9894 individuals had data available from at least 6 of the 12 time points and the model fit parameters were broadly consistent with the smaller but more complete dataset (Table E1 and Fig E1b). The entropies for the 4- and 6-class models were 0.78 and 0.75, respectively indicating only small decreases in model fit compared to the smaller, but complete analysis and the individual classifications for each class, were all >0.7, with <0.13 for all off-diagonals. Comparison of results from the larger incomplete and smaller but complete datasets showed similar class assignment: only 3% of children changed best-fit class between the 6-class models in each analysis (Table E2) and the proportions of individuals in each class are very similar between models (Table E3)

Moving from the 6-class to the 4-class model, 99% of the persistent class (733/739) and the normal class (6111/6168) remained in these classes in the 4 class model. The majority of the early-onset-early-resolving (81%) and early-onset-late-resolving (61%) were in the 4-class early-onset class. The majority of the mid-onset (49%) and late-onset (87%) were in the 4-class late-onset class (Table E4).

PIAMA – model fit

In the dataset of complete data (N=2063), the BIC was lowest for a 4 class model (Table E5 & Fig E2a) and the VLMR LR p-value showed evidence of improved fit for the 4-class model compared to a 3-class model (p=0.015). However, there was only a small increase in BIC in the 6 class model (Table E5 & Fig E2a).

3652 individuals had data available from at least 5 of the 10 time points and the model fit parameters were broadly consistent with the smaller but more complete dataset (Table E5 and Fig E2b). The entropies for the 4- and 6-class models were 0.76 and 0.81, respectively indicating only small decreases in model fit compared to the smaller, but complete analysis and the proportions of individuals in each class are similar between the incomplete and complete analyses (Table E6).

Moving from the 6-class to the 4-class model, similar to ALSPAC, 99% of the persistent class (178/180) and the normal class (2468/2497) remained in these classes in the 4 class model. The majority of the early-onset-early-resolving (51%) and early-onset-late-resolving (68%) were in the 4-class early-onset class. The majority of the mid-onset (55%) and late-onset (91%) were in the 4-class late-onset class (Table E7).

Average latent class probability assignments

Average latent class probability assignments were >70% for all classes in the ALSPAC and PIAMA final models (Table E8). Unaffected and persistent classes had the highest probabilities (>80% in all cases). The largest probabilities for alternative class membership were between persistent and early-onset later-resolving in ALSPAC (12%) and early-onset early-resolving an unaffected in PIAMA (18%).

Bias –adjusted three step method for testing association of covariates with latent classes.

Association of risk factors and comorbidities with the latent classes were tested using a bias-adjusted three step analysis^9^ to account for uncertainty in class assignment.

Following the LLCA (step 1), class assignment probabilities were exported to Stata version 14-MP^10^ and logit constraints were derived to define the relationship between modal (or best) class (W) and latent classes (X) (which account for the uncertainty in modal class) (step 2). These logit constraints were then used in regression analyses in MPlus (step 3).

**Supplementary References**

1. Boyd A., Golding J., Macleod J., Lawlor D.A., Fraser A., Henderson J. *et al*. Cohort Profile: the 'children of the 90s'--the index offspring of the Avon Longitudinal Study of Parents and Children. Int J Epidemiol 42, 111-27 (2013).

2. Fraser A., Macdonald-Wallis C., Tilling K., Boyd A., Golding J., Davey Smith G., *et al*. Cohort Profile: the Avon Longitudinal Study of Parents and Children: ALSPAC mothers cohort. Int J Epidemiol 42, 97-110 (2013).

3. http://www.bris.ac.uk/alspac/researchers/data-access/data-dictionary/.

4. He, C., Holme, J. & Anthony, J. SNP genotyping: the KASP assay. Methods Mol Biol 1145, 75-86 (2014).

5. Paternoster L., Standl M. Waage J., Baurecht H., Hotze M., Strachan D.P. *et al*. Multi-ancestry genome-wide association study of 21,000 cases and 95,000 controls identifies new risk loci for atopic dermatitis. Nat Genet 47, 1449-56 (2015).

6. Wijga A.H., Kerkhof M., Gehring U., de Jongste J.C., Postma D.S., Aalberse R.C. *et al*. Cohort profile: the prevention and incidence of asthma and mite allergy (PIAMA) birth cohort. Int J Epidemiol 43, 527-35 (2014).

7. Bottema R.W., Reijmerink N.E., Kerkhof M., Koppelman G.H., Stelma F.F., Gerritsen J. *et al*. Interleukin 13, CD14, pet and tobacco smoke influence atopy in three Dutch cohorts: the allergenic study. Eur Respir J 32, 593-602 (2008).

8. Moffatt M.F., Gut I.G., Strachan D.P., Bouzigon E., Heath S., von Mutius E. *et al*. A large-scale, consortium-based genomewide association study of asthma. N Engl J Med 363, 1211-21 (2010).

9. Heron J.C., Croudace T.J., Barker E.D., Tilling K. A comparison of approaches for assessing covariate effects in latent class analysis. Longitudinal and Life Course Studies 6, 420-434 (2015).

10. StataCorp. Stata Statistical Software: Release 14. (StataCorp LP., College Station, TX, 2015).

**Figure Legends**

**Fig E1. Bayesian Information Criterion for ALSPAC model fit**. (a) smaller dataset with complete data (N=3480), (b) larger dataset with ≥6 of the 12 time-points available (N=9894).

**Fig E2. Bayesian Information Criterion across all PIAMA models**, for (a) individuals with complete data (N=2063), and (b) individuals with incomplete data (N=3652)

**Fig E3. 4-class model in ALSPAC**

**Fig E4. 4-class model in PIAMA**

**Tables**

**Table E1.** **LLCA model fit parameters in the ALSPAC cohort**

|  | Individuals with complete data (N=3480) | | |  | Individuals with ≥6/12 time points available (N=9894) | | |
| --- | --- | --- | --- | --- | --- | --- | --- |
| Number of classes | BIC | Entropy | VLMR LRT  p-value |  | BIC | Entropy | VLMR LR p-value |
| 2 | 33643 | 0.91 | <0.0001 |  | 78475 | 0.88 | <0.0001 |
| 3 | 32580 | 0.83 | <0.0001 |  | 76525 | 0.79 | <0.0001 |
| **4** | **32182** | **0.83** | **<0.0001** |  | **75655** | **0.78** | **<0.0001** |
| 5 | 32083 | 0.80 | 0.0394 |  | 75386 | 0.77 | <0.0001 |
| **6** | **32035** | **0.80** | **0.0177** |  | **75288** | **0.75** | **0.1289** |
| 7 | 32049 | 0.80 | 0.0449 |  | 75212 | 0.75 | 0.0001 |

BIC, Bayesian Information Criterion; VLMR LRT, Vuong–Lo–Mendell–Rubin Likelihood ratio test. The 4- and 6-class models (**in bold**), both showed evidence of good model fit; the 6-class model had the lowest BIC value, but this was only a minor improvement in model fit compared to the 4-class model, according to VLMR LRT.

**Table E2. Comparison of ALSPAC 6 class models constructed with individuals with complete data and constructed with individuals with incomplete data**

| incomplete  complete | P | EO-LR | EO-ER | MO-R | LO-R | unaffected |
| --- | --- | --- | --- | --- | --- | --- |
| P | 282 | 0 | 0 | 0 | 1 | 0 |
| EO-LR | 0 | 264 | 4 | 8 | 0 | 0 |
| EO-ER | 0 | 7 | 387 | 20 | 4 | 26 |
| MO-R | 5 | 0 | 0 | 193 | 3 | 1 |
| LO-R | 0 | 2 | 4 | 6 | 246 | 17 |
| unaffected | 0 | 0 | 0 | 6 | 2 | 1992 |

Incomplete data represents at least 6 of the 12 time-points available; the majority of individuals (diagonals, 97%) do not change classes; 116/3480 (3%) change classes.

In order to carry out this comparison individuals were assigned to their most likely class for the two models. Given that entropies are only 0.75-0.8, this is an approximation.

**Table E3. Comparison of proportion of individuals in ALSPAC 6 class models constructed with complete data and constructed with individuals with incomplete data**

| Class | Incomplete analysis | Complete analysis |
| --- | --- | --- |
| P | 7.3% | 7.9% |
| EO-LR | 7.0% | 8.1% |
| EO-ER | 12.9% | 14.5% |
| MO-R | 7.0% | 6.0% |
| LO-R | 7.9% | 8.7% |
| unaffected | 58.0% | 54.9% |

**Table E4.** **Comparison of ALSPAC 4- & 6-class models constructed with individuals with at least 6 of the 12 time-points available.**

| 4 class  6 class | P | EO-R | LO-R | unaffected |
| --- | --- | --- | --- | --- |
| P | 733 | 2 | 4 | 0 |
| EO-LR | 246 | 401 | 11 | 0 |
| EO-ER | 0 | 876 | 2 | 206 |
| MO-R | 64 | 238 | 296 | 3 |
| LO-R | 12 | 23 | 545 | 45 |
| unaffected | 0 | 14 | 43 | 6111 |

In order to carry out this comparison individuals were assigned to their most likely class for the two models. Given that entropies are only 0.75-0.78, this is an approximation.

**Table E5.** **LLCA model fit parameters in the PIAMA cohort**

|  | Individuals with complete data (N=2063) | | |  | Individuals with ≥5/10 time points available (N=3652) | | |
| --- | --- | --- | --- | --- | --- | --- | --- |
| Classes | BIC | Entropy | VLMR LR p-value |  | BIC | Entropy | VLMR LR p-value |
| 2 | 14395 | 0.92 | <0.0001 |  | 23850 | 0.91 | <0.0001 |
| 3 | 14147 | 0.83 | <0.0001 |  | 23476 | 0.82 | <0.0001 |
| **4** | **14067** | **0.84** | **0.0146** |  | **23327** | **0.81** | **0.0139** |
| 5 | 14076 | 0.76 | 0.0888 |  | 23303 | 0.76 | 0.0675 |
| **6** | **14105** | **0.73** | **0.0228** |  | **23312** | **0.76** | **0.0751** |
| 7 | 14141 | 0.71 | 0.3658 |  | 23343 | 0.78 | 0.0057 |

**Table E6. Comparison of proportion of individuals in PIAMA 6 class models constructed with complete data and constructed with individuals with incomplete data**

| Class | Incomplete analysis | Complete analysis |
| --- | --- | --- |
| P | 4.9% | 4.8% |
| EO-LR | 3.8% | 4.5% |
| EO-ER | 15.4% | 12.4% |
| MO-R | 6.5% | 8.7% |
| LO-R | 6.5% | 11.8% |
| unaffected | 62.9% | 57.8% |

**Table E7. Comparison of PIAMA 4 & 6 class models constructed with individuals with at least 5 of the 10 time-points available**.

| 4 class  6 class | P | EO-R | LO-R | unaffected |
| --- | --- | --- | --- | --- |
| P | 178 | 0 | 2 | 0 |
| EO-LR | 44 | 98 | 2 | 0 |
| EO-ER | 0 | 203 | 6 | 191 |
| MO-R | 26 | 76 | 126 | 0 |
| LO-R | 1 | 5 | 184 | 13 |
| unaffected | 0 | 0 | 29 | 2468 |

In order to carry out this comparison individuals were assigned to their most likely class for the two models. Given that entropies are only 0.76-0.81, this is an approximation.

**Table E8. Average latent class probabilities for the most likely class membership (row) by latent class (column).**

|  | ALSPAC | |  |  |  |  |  | PIAMA |  |  |  | | |  | |  |
| --- | --- | --- | --- | --- | --- | --- | --- | --- | --- | --- | --- | --- | --- | --- | --- | --- |
|  | P | EO-LR | EO-ER | MO-R | LO-R | UA |  | P | EO-LR | EO-ER | MO-R | LO-R | UA | |  |  |
| P | 0.834 | 0.122 | 0.004 | 0.030 | 0.009 | 0.000 |  | 0.902 | 0.047 | 0.000 | 0.042 | 0.009 | 0.000 | |  |  |
| EO-LR | 0.093 | 0.718 | 0.094 | 0.076 | 0.019 | 0.001 |  | 0.042 | 0.764 | 0.092 | 0.075 | 0.025 | 0.002 | |  |  |
| EO-ER | 0.004 | 0.069 | 0.735 | 0.047 | 0.047 | 0.098 |  | 0.001 | 0.031 | 0.718 | 0.032 | 0.043 | 0.175 | |  |  |
| MO-R | 0.042 | 0.048 | 0.070 | 0.716 | 0.081 | 0.043 |  | 0.03 | 0.022 | 0.081 | 0.739 | 0.084 | 0.044 | |  |  |
| LO-R | 0.016 | 0.023 | 0.061 | 0.097 | 0.722 | 0.082 |  | 0.02 | 0.012 | 0.066 | 0.083 | 0.717 | 0.103 | |  |  |
| UA | 0.000 | 0.001 | 0.054 | 0.012 | 0.034 | 0.898 |  | 0.000 | 0.000 | 0.092 | 0.008 | 0.021 | 0.879 | |  |  |

UA=unaffected
